# Atherogenic Risk and Its Association with Alcohol Consumption, Lifestyle Habits, and Sociodemographic Factors in a Population of Spanish Workers

**DOI:** 10.3390/life15060923

**Published:** 2025-06-07

**Authors:** Joan Obrador de Hevia, Ángel Arturo López-González, José Ignacio Ramírez-Manent, Carla Busquets-Cortes, Pedro Juan Tárraga López, Miguel García Samuelsson, Pere Riutord-Sbert

**Affiliations:** 1 ADEMA-Health Group of IUNICS, 07009 Palma de Mallorca, Balearic Islands, Spain; j.obrador@eua.edu.es (J.O.d.H.); jignacioramirez@telefonica.net (J.I.R.-M.); c.busquets@eua.edu.es (C.B.-C.); miguelsamuelsson@gmail.com (M.G.S.); pereriutord@gmail.com (P.R.-S.); 2 Primary Care in Mallorca, 07181 Palma de Mallorca, Balearic Islands, Spain; 3 Faculty of Medicine, University of Castilla la Mancha, 02008 Albacete, Albacete, Spain; pjtarraga@sescam.jccm.es

**Keywords:** atherogenic dyslipidemia, atherogenic risk, physical activity, Mediterranean diet, alcohol consumption, socioeconomic status

## Abstract

Atherogenic dyslipidemia is a major contributor to cardiovascular disease, the leading cause of morbidity and mortality worldwide. While lipid abnormalities are well-established clinical risk factors, growing evidence highlights the influence of sociodemographic and lifestyle determinants on lipid profiles. However, large-scale epidemiological data addressing these associations within occupational settings remain limited. **Objective:** To assess the cross-sectional and longitudinal associations between atherogenic risk—measured through TC/HDL-c, LDL-c/HDL-c, TG/HDL-c ratios, and atherogenic dyslipidemia (AD)—and sociodemographic variables, health behaviors, and alcohol consumption in a large cohort of Spanish workers. **Methods:** A dual-phase study was conducted. The first phase was a cross-sectional analysis of 139,634 workers (83,282 men; 56,352 women) from multiple employment sectors undergoing routine occupational health assessments. The second phase was a longitudinal study of a subsample (*n* = 40,431) with complete data from 2009 and 2019. Clinical, anthropometric, and biochemical data were collected using standardized protocols. Lifestyle factors (smoking, physical activity, Mediterranean diet adherence, alcohol intake) and socioeconomic indicators (education, occupational class) were recorded. Multinomial logistic regression was used to determine independent associations with high-risk atherogenic profiles. **Results:** Higher atherogenic indices and prevalence of AD were associated with advancing age, lower educational level, lower social class, smoking, physical inactivity, poor diet quality, and alcohol consumption. Men exhibited higher TG/HDL-c and AD values, whereas women had higher TC/HDL-c and LDL-c/HDL-c. Physical inactivity showed the strongest association with TG/HDL-c (OR: 36.23; 95% CI: 32.12–40.35) and AD (OR: 16.86; 95% CI: 14.80–18.93). Alcohol intake also independently predicted higher TG/HDL-c (OR: 1.60) and AD (OR: 1.79). Over the decade, a general increase in atherogenic risk was observed, especially among older adults, socially disadvantaged groups, and those with unhealthy behaviors. **Conclusions:** Sociodemographic and lifestyle factors, particularly physical inactivity and alcohol consumption, are strongly associated with adverse atherogenic profiles in the working population. The observed rise in lipid-related cardiovascular risk over the past decade emphasizes the urgent need for workplace-based health promotion strategies targeting modifiable behaviors and structural health inequalities.

## 1. Introduction

Cardiovascular disease (CVD) remains the leading cause of morbidity and mortality worldwide, accounting for an estimated 17.9 million deaths annually and posing a substantial burden on healthcare systems and economies alike [1]. Atherogenic dyslipidemia, characterized by elevated triglycerides, low high-density lipoprotein cholesterol (HDL-c), and small, dense low-density lipoprotein cholesterol (LDL-c) particles, is a central component of the pathophysiological cascade that underpins atherosclerosis and subsequent cardiovascular events [2,3]. The identification of individuals at increased atherogenic risk through routine biochemical profiling is therefore a critical component of both primary and secondary prevention strategies.

The clinical utility of lipid ratios—such as total cholesterol to HDL-c (TC/HDL-c), LDL-c to HDL-c (LDL-c/HDL-c), and triglycerides to HDL-c (TG/HDL-c)—has garnered increasing attention as surrogate markers for assessing atherogenic risk beyond traditional lipid parameters [4,5,6]. These composite indices integrate pro-atherogenic and anti-atherogenic lipid fractions, thus providing a more robust estimate of residual cardiovascular risk, particularly in populations with metabolic disturbances such as obesity, insulin resistance, and type 2 diabetes [7,8,9]. Among these, the TG/HDL-c ratio has been widely recognized as a sensitive marker of insulin resistance and a predictor of cardiovascular events, even in normolipidemic individuals [10,11].

While the biological determinants of dyslipidemia are well-established, there is growing recognition of the critical role that sociodemographic and behavioral factors play in modulating lipid profiles and associated cardiovascular risk [12,13]. Health behaviors—including dietary patterns, physical activity, smoking, and alcohol consumption—are amenable to modification and are influenced by broader social determinants such as education, income, and occupational class [14,15,16]. These factors operate synergistically and often unequally across population subgroups, contributing to disparities in cardiovascular health outcomes.

Among lifestyle factors, adherence to the Mediterranean diet—a dietary pattern rich in fruits, vegetables, whole grains, legumes, nuts, olive oil, and moderate wine intake—has been associated with favorable lipid profiles and reduced incidence of cardiovascular events [17,18]. Conversely, low physical activity levels and smoking are consistently linked to atherogenic lipid patterns and higher rates of coronary heart disease [19,20]. The role of alcohol consumption, however, remains more contentious. While moderate alcohol intake has been associated with cardioprotective effects in some studies, largely attributed to its impact on HDL-c and anti-inflammatory pathways [21,22], emerging evidence suggests that any potential benefits may be outweighed by its adverse effects on triglyceride levels, blood pressure, and overall mortality risk, particularly when intake exceeds recommended limits [23,24,25].

In parallel, sociodemographic gradients—such as educational attainment and occupational class—also influence lipid metabolism, either directly through physiological stress pathways or indirectly via access to resources, health literacy, and opportunities for healthy behavior [26,27]. Individuals with lower socioeconomic status are more likely to experience chronic stress, poor diet quality, limited access to preventive healthcare, and increased exposure to obesogenic environments, all of which may contribute to a pro-atherogenic lipid profile [28,29,30].

Despite this growing body of evidence, there remains a paucity of large-scale epidemiological studies examining the combined and interactive effects of lifestyle and sociodemographic variables on atherogenic risk, particularly in working populations. Most prior research has focused on clinical cohorts or general populations, often with limited representation of occupational strata and behavioral diversity. The occupational context, however, provides a unique opportunity to assess the influence of work-related and lifestyle factors on cardiovascular health, given the structured environment, potential for workplace interventions, and the critical role of working-age individuals in the productivity and sustainability of health systems.

To address these gaps, the present study investigates the association between atherogenic risk, health behaviors, and sociodemographic variables in a large and diverse cohort of 139,634 Spanish workers. Using standardized clinical, anthropometric, and lifestyle assessments collected through a structured workplace health surveillance program, we examine the cross-sectional and longitudinal trends of three key atherogenic indices—TC/HDL-c, LDL-c/HDL-c, and TG/HDL-c—as well as the presence of atherogenic dyslipidemia (AD), a composite indicator defined by elevated triglycerides and reduced HDL-c levels. Our objective is twofold: (1) to identify sociodemographic and lifestyle determinants independently associated with high-risk atherogenic profiles, and (2) to explore sex-specific patterns and temporal trends over a ten-year period.

This study builds upon the existing literature by integrating a wide range of individual-level variables—including age, sex, social class, education, smoking, physical activity, dietary adherence, and alcohol consumption—into a comprehensive multivariate model. The use of routinely collected occupational health data ensures external validity and real-world applicability, while the large sample size enhances statistical power and permits detailed stratified analyses. Furthermore, the inclusion of a longitudinal component allows for an assessment of temporal changes in risk patterns, which is particularly relevant in the context of evolving public health policies and lifestyle trends in Spain over the last decade.

The relevance of this study is underscored by recent epidemiological trends indicating a resurgence in metabolic disorders, including obesity and dyslipidemia, in many European countries despite advances in medical therapy and public health awareness [31,32]. In Spain, where the Mediterranean lifestyle has traditionally been considered protective, the erosion of healthy habits in the face of urbanization, sedentary work environments, and increased alcohol intake among certain subgroups may be reversing prior gains in cardiovascular risk reduction [33,34,35]. Understanding how these sociodemographic and behavioral changes translate into biochemical risk profiles is essential for guiding targeted prevention efforts and resource allocation.

Moreover, the occupational health setting represents a strategic entry point for the implementation of cardiovascular risk screening and lifestyle interventions. Employers and policymakers can leverage the findings of this study to design tailored health promotion programs aimed at high-risk subgroups, address structural determinants of unhealthy behavior, and foster environments conducive to cardiovascular well-being. By identifying modifiable risk factors linked to atherogenic dyslipidemia in working adults, this research contributes to the broader agenda of reducing premature cardiovascular mortality and achieving equity in health outcomes.

In summary, the present investigation offers a robust epidemiological analysis of the associations between alcohol consumption, other health-related behaviors, sociodemographic factors, and atherogenic risk profiles in a large occupational cohort in Spain. Through the use of validated lipid ratios, comprehensive data collection, and advanced statistical modeling, we aim to elucidate the interplay between lifestyle, social determinants, and biochemical risk, providing actionable insights for clinical practice, public health policy, and future research in preventive cardiology.

## 2. Methods

### 2.1. Study Design and Population

This investigation was structured in two distinct phases. The first consisted of a large-scale, cross-sectional descriptive analysis involving 139,634 employed individuals from diverse geographic regions across Spain, encompassing nearly all professional sectors (83,282 men and 56,352 women). Participants were selected from those undergoing routine occupational health examinations within affiliated companies.

In the second phase, a retrospective longitudinal analysis was conducted on a subsample of 40,431 individuals (24,229 men and 16,202 women) who had complete clinical records spanning from 2009 to 2019. This cohort was derived from the original population and met specific inclusion criteria to ensure data consistency over time.

All anthropometric, clinical, and laboratory data were collected by qualified healthcare professionals within the occupational health units of the participating organizations. Standardized protocols were strictly followed to minimize inter-observer variability and ensure data reliability.

The eligibility criteria for inclusion in the study were as follows:

- Age between 18 and 69 years, corresponding to the active working population;
- Active employment status at the time of assessment, without any recorded temporary incapacity;
- Availability of complete data to compute the various atherogenic risk scores;
- Provision of informed consent authorizing the use of anonymized data for epidemiological research purposes;
- For the longitudinal component, availability of comprehensive data for both 2009 and 2019, and no recorded changes in sociodemographic attributes or health behaviors during this interval.

**Figure 1:**
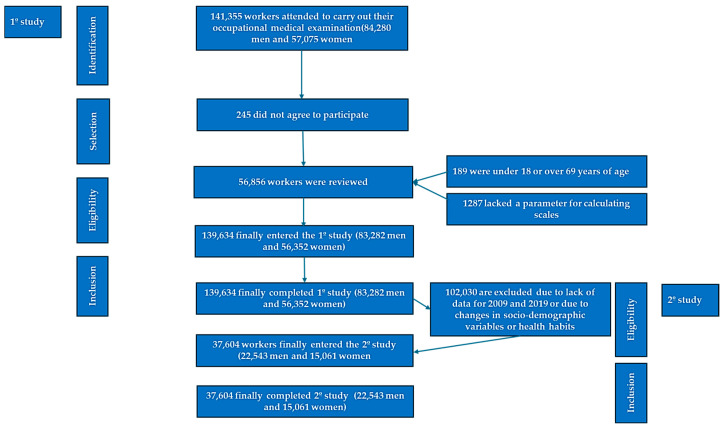
Flow chart of the participants.

The participant selection methodology is summarized in the flow diagram provided in Figure 1.

### 2.2. Variable Assessment

Clinical, biochemical, and anthropometric measurements were performed by trained personnel adhering to uniform procedures. Waist circumference was measured using a non-elastic tape placed at the level of the last rib, with the subject standing in a relaxed position. Blood pressure was assessed with an OMRON M3 sphygmomanometer (OMRON, Osaka, Japan), following ten minutes of seated rest. Three consecutive readings were obtained, and the mean value was recorded for analysis.

Venous blood samples were drawn following a 12 h fasting period. Serum HDL cholesterol was determined via precipitation techniques, while glucose, triglycerides, and total cholesterol were quantified using enzymatic colorimetric assays. LDL cholesterol was calculated using the Friedewald formula. All biochemical values were reported in mg/dL.

Risk classification thresholds for the lipid ratios were defined as follows [36]:

- Total cholesterol/HDL-c: Low risk if <5 in men and <4.5 in women; moderate risk between 5 and 9 in men and 4.5 and 7 in women; high risk if >9 in men and >7 in women;
- LDL-c/HDL-c: Values < 3 were categorized as low risk, while values ≥ 3 were considered high risk;
- Triglycerides/HDL-c: A ratio ≥ 3 indicated elevated risk.

Atherogenic dyslipidemia (AD) was defined by the simultaneous presence of triglyceride levels > 150 mg/dL, reduced HDL-c (<40 mg/dL in men and <50 mg/dL in women), and normal LDL-c values [37].

Occupational social class was determined in accordance with the 2011 National Classification of Occupations (CNO-11) and stratified based on the criteria of the Spanish Society of Epidemiology into three categories [38]:

- Class I: Professionals and senior management;
- Class II: Self-employed and intermediate-skilled workers;
- Class III: Manual and unskilled labor.

Educational attainment was recorded at three levels: primary education, secondary education, and tertiary (university-level) education.

Smoking status was categorized based on current or recent tobacco use. Individuals who reported smoking at least one cigarette daily (or an equivalent form of tobacco) within the past 30 days or who had quit smoking within the preceding 12 months were classified as smokers.

Dietary habits were assessed using the validated PREDIMED Mediterranean Diet Adherence Questionnaire, consisting of 14 dichotomous items (0 or 1). A score of 9 or higher denoted strong adherence to the Mediterranean dietary pattern, recognized for its cardioprotective properties [39].

Physical activity was evaluated using the International Physical Activity Questionnaire (IPAQ), which captures data on the frequency and duration of physical activity during the previous week [40].

Alcohol intake was measured in standard alcohol units (SAUs), with one SAU equivalent to 10 g of pure ethanol. According to national guidelines (Spanish Ministry of Health, 2020) [41], low-risk alcohol consumption thresholds are set at 20 g/day for men and 10 g/day for women, acknowledging that there is no zero-risk level. High alcohol consumption was defined as an intake of ≥14 SAUs per week for women and ≥21 SAUs per week for men [42].

### 2.3. Statistical Analysis

Continuous variables were analyzed using the Student’s *t*-test to compare mean differences, while categorical variables were assessed using the chi-square test. The normality of continuous variables was assessed using the Kolmogorov–Smirnov test. Variables that met normality assumptions were analyzed using the Student’s *t*-test. Multivariate analyses were conducted using a multinomial logistic regression model to identify independent associations between explanatory variables and atherogenic risk profiles. Statistical significance was defined by a *p*-value of <0.05. All statistical computations were performed using SPSS software, version 28.0 (IBM Corp., Armonk, NY, USA).

## 3. Results

Table 1 presents significant gender differences across all anthropometric, clinical, and lifestyle variables. Men showed higher values for weight, height, blood pressure, total cholesterol, LDL-c, triglycerides, and glucose, while women exhibited higher HDL-c levels. These findings are consistent with known gender-based physiological differences. Additionally, the distribution of social class, educational attainment, and health behaviors differed markedly between the sexes. A higher proportion of women reported adherence to healthy habits such as physical activity, Mediterranean diet, and abstention from alcohol, which may partially explain their more favorable lipid profiles.

The data demonstrate clear trends in the atherogenic indices (TC/HDL-c, LDL-c/HDL-c, and TG/HDL-c) across age, social class, education, and health behaviors. A progressive increase in atherogenic ratios with age is evident for both sexes, particularly pronounced in TG/HDL-c. Socioeconomic disparities are notable: individuals with higher education and higher social class tend to present lower mean values for all indices. Healthy behaviors such as non-smoking, physical activity, adherence to the Mediterranean diet, and alcohol abstinence are consistently associated with lower atherogenic indices, highlighting the modifiable nature of these risk factors (Table 2).

The prevalence of high-risk atherogenic profiles increases with age in both men and women, reaching the highest levels among individuals aged 60–69 years. Lower educational level and social class III are associated with a greater proportion of participants exhibiting elevated TC/HDL-c, LDL-c/HDL-c, TG/HDL-c, and AD (atherogenic dyslipidemia). Notably, physical inactivity and poor adherence to the Mediterranean diet are strongly linked to a higher prevalence of all risk scales, especially among men. Alcohol consumption is also related to increased atherogenic risk, particularly TG/HDL-c and AD, reinforcing the role of lifestyle in cardiometabolic health (Table 3).

The regression analysis confirms the univariate findings and quantifies the associations. Male sex was associated with lower odds for high TC/HDL-c and LDL-c/HDL-c but a markedly higher risk for TG/HDL-c (OR = 3.76) and AD (OR = 1.84), indicating a more atherogenic lipid pattern linked to hypertriglyceridemia. Advancing age, lower educational level, social class III, smoking, physical inactivity, poor diet quality, and alcohol consumption were all independent predictors of high-risk values across the atherogenic scales. Physical inactivity and alcohol use showed particularly strong associations with TG/HDL-c (OR = 36.23 and OR = 1.60, respectively) and AD (OR = 16.86 and OR = 1.79, respectively), emphasizing their key roles as intervention targets (Table 4).

Table 5 provides a decade-long retrospective analysis (2009–2019) of changes in the prevalence of moderate to high values of various atherogenic risk indicators—TC/HDL-c, LDL-c/HDL-c, TG/HDL-c ratios, and atherogenic dyslipidemia (AD)—disaggregated by sex and stratified by age, social class, educational level, and lifestyle behaviors. The findings offer valuable insights into temporal dynamics in cardiovascular risk among a large occupational cohort.

Overall, the data reveal a generalized increase in atherogenic risk across most strata, with higher relative increases observed in older age groups, individuals of lower socioeconomic status, and those exhibiting unhealthy behaviors. Notably, the most pronounced percentage increases were seen in men aged 50–59 years and women aged 60–69 years, where AD rose by 17.5% and 14.8%, respectively. These findings align with the well-established age-related rise in dyslipidemia and its contribution to cardiometabolic risk.

From a sociodemographic perspective, both men and women from social class III and with only elementary education experienced markedly higher increases in all risk markers compared to their counterparts with university education or higher social status. For example, men with elementary education showed an 18.1% increase in TC/HDL-c and a 17.1% rise in AD, while women in the same category exhibited increases of 18.1% and 14.3%, respectively. This underscores the persistent health inequalities driven by social determinants of health.

In terms of lifestyle factors, physical inactivity and poor adherence to the Mediterranean diet were consistently associated with the greatest increases across all risk indices. Men who did not engage in physical activity experienced a 19.6% increase in AD, while inactive women had an 11.0% increase. Similarly, among men not following a Mediterranean diet, TG/HDL-c increased by 20.1%, and AD rose by 13.1%; comparable trends were observed in women. These results highlight the modifiable nature of these risk factors and reinforce the value of promoting sustained lifestyle interventions in the workplace.

Interestingly, alcohol consumption was also linked to adverse changes in lipid profiles over time. Men who consumed alcohol exhibited a 19.8% increase in AD, and women exhibited a 17.2% increase, both significantly higher than those who abstained. While moderate alcohol intake is sometimes viewed as cardioprotective, these data suggest that, in this population, alcohol consumption may exacerbate lipid-related cardiovascular risk, particularly in conjunction with other unhealthy behaviors.

Importantly, individuals reporting healthier behaviors, such as physical activity and adherence to the Mediterranean diet, demonstrated only marginal increases—or even stabilization—in atherogenic indices over the decade. For instance, physically active men saw only a 7.1% increase in AD compared to 23.8% in their inactive peers, while women adhering to the Mediterranean diet showed a modest 5.9% increase in AD, compared to 14.6% in those who did not.

Taken together, the findings in Table 5 emphasize the critical influence of both social and behavioral factors on long-term atherogenic risk. The stratified analysis provides robust evidence that targeted public health interventions, particularly those addressing physical inactivity, poor dietary habits, and socioeconomic disparities, could yield substantial benefits in mitigating cardiovascular risk in occupational populations.

**Figure 2:**
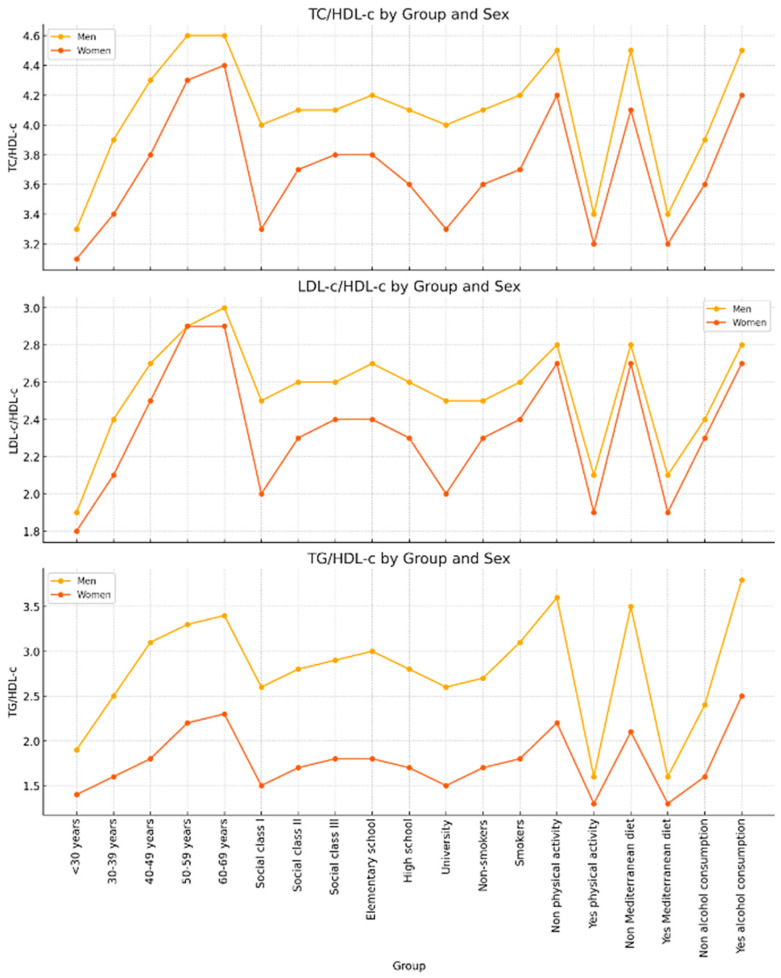
The graph shows the TC/HDL-c, LDL/HDL-c, and TG/HDL-c ratios broken down by group and sex.

Figure 2 shows the TC/HDL-c, LDL/HDL-c, and TG/HDL-c ratios stratified by group and sex, allowing for the analysis of differences in lipid profiles between men and women according to age, social class, educational level, and lifestyle factors. Overall, men exhibit higher values across all indices, particularly for the TG/HDL-c ratio, suggesting a more atherogenic lipid profile in this group. A progressive increase in these ratios is observed with age up to 50–59 years, followed by a slight stabilization or decline. Men from lower social classes (class III) and with lower educational attainment (elementary school) present the highest TC/HDL-c and TG/HDL-c ratios. Lifestyle factors also play a significant role: smokers, physically inactive individuals, and those with low adherence to the Mediterranean diet or higher alcohol consumption show markedly elevated values, with notable sex differences. In particular, the TG/HDL-c ratio reaches its highest values in men with low physical activity and poor adherence to the Mediterranean diet. This pattern suggests that lifestyle-focused interventions could have a significant impact on lipid profiles, especially among men and socioeconomically vulnerable subgroups.

## 4. Discussion

This study examined the associations between sociodemographic characteristics, lifestyle behaviors, and atherogenic risk indicators—including TC/HDL-c, LDL-c/HDL-c, TG/HDL-c ratios, and atherogenic dyslipidemia (AD)—in a large cohort of over 139,000 Spanish workers. The findings provide compelling evidence that age, sex, educational level, occupational class, smoking status, physical activity, dietary pattern, and alcohol consumption are all independently associated with unfavorable atherogenic profiles. Moreover, the longitudinal analysis reveals a worsening trend in lipid-related cardiovascular risk over the last decade, particularly among those in lower socioeconomic strata and those with unhealthy behaviors.

Consistent with previous research, we observed that atherogenic indices increased progressively with age, with the highest values and prevalence of AD found in the oldest age group (60–69 years). This age-related rise in atherogenic burden is likely due to cumulative exposure to cardiometabolic risk factors, hormonal changes, and endothelial dysfunction. Notably, men presented with significantly higher mean values and prevalence of high TG/HDL-c and AD despite having lower odds for high TC/HDL-c and LDL-c/HDL-c compared to women. These sex-specific differences may reflect variations in fat distribution, insulin sensitivity, and hormonal influences on lipid metabolism [43]. The triglyceride-to-high-density-lipoprotein-cholesterol ratio (TG/HDL-c) is a widely accepted surrogate marker of insulin resistance (IR) and cardiometabolic risk. However, its optimal cut-off values vary across populations due to genetic, metabolic, lifestyle, and environmental differences. In the Spanish population, cut-off points between 2.4 and 3.0 have been associated with increased IR and metabolic syndrome risk, particularly in adults without diagnosed diabetes. These thresholds are generally lower than those reported in the United States, where values ≥ 3.5 or ≥4.0 are often used, especially in populations with higher obesity and type 2 diabetes prevalence [44]. Ethnic differences further complicate the establishment of universal TG/HDL-c cut-off points. Non-Hispanic Black individuals typically exhibit lower triglyceride and higher HDL-c levels than non-Hispanic Whites and Mexican Americans, resulting in lower TG/HDL-c ratios despite similar or higher insulin resistance. Consequently, applying a uniform threshold across diverse populations may lead to risk misclassification and underscores the need for population-specific reference values [45,46].

Moreover, sex-specific differences have been observed. A comprehensive review of 32 studies involving nearly 50,000 participants reported average TG/HDL-c cut-off values of 2.53 for women and 2.8 for men, highlighting the necessity for gender-specific thresholds [47]. These variations highlight the need to establish population-specific reference values for the TG/HDL-c ratio. Using cut-off points derived from one population may not be appropriate for another and could lead to under- or overestimation of metabolic risk. Therefore, clinicians and researchers should consider ethnic, regional, and sex-specific factors when interpreting this index. In conclusion, while the TG/HDL-c ratio is a valuable and accessible marker for assessing insulin resistance and cardiometabolic risk, its clinical utility improves when tailored to specific populations. Future research should aim to refine these thresholds using large, representative cohorts stratified by ethnicity, sex, and other relevant factors to enhance risk stratification and guide personalized prevention strategies.

From a sociodemographic standpoint, participants with lower educational attainment and those in social class III consistently exhibited higher mean values and prevalence of high-risk atherogenic indices. These findings align with the well-established relationship between socioeconomic disadvantage and cardiovascular risk and likely reflect underlying disparities in access to health information, healthcare services, and the adoption of healthy behaviors. Importantly, the differences persisted even after adjustment for behavioral factors, suggesting a residual effect of social determinants on lipid metabolism, possibly mediated through chronic stress or occupational exposures [47].

The analysis of lifestyle behaviors revealed strong associations between unhealthy habits and atherogenic risk. Physical inactivity emerged as the most potent behavioral predictor of all atherogenic indices, particularly TG/HDL-c and AD, with odds ratios as high as 36.23 and 16.86, respectively. These findings are supported by the extensive literature linking sedentary behavior with insulin resistance, hypertriglyceridemia, and low HDL-c levels [48]. Similarly, poor adherence to the Mediterranean diet was associated with significantly increased risk, reinforcing the protective role of this dietary pattern in cardiovascular health.

Alcohol consumption, often debated in the context of cardiovascular risk, was consistently associated with worse lipid profiles. Individuals who reported alcohol intake had higher values across all atherogenic scales and a greater likelihood of presenting with AD. High-risk alcohol consumption is defined differently across countries, reflecting variations in cultural norms, public health policies, and epidemiological criteria. In Spain, national guidelines define high-risk intake as ≥14 standard alcohol units (SAUs) per week for women and ≥21 SAUs for men, with one SAU equivalent to 10 g of pure ethanol (Spanish Ministry of Health, 2020 [40]). While this aligns with recommendations from some European countries, thresholds and unit definitions vary.

In the United Kingdom, guidelines advise both men and women not to exceed 14 units per week, with each unit containing 8 g of ethanol (U.K. Department of Health, 2016) [49], resulting in a lower maximum intake, particularly for men. Germany recommends no more than 12 g/day for women and 24 g/day for men (BZgA, 2020) [50], and France sets a limit of 10 standard drinks per week, each containing approximately 10 g of ethanol (Santé Publique France, 2019) [51].

In the United States, moderate consumption is defined as up to one standard drink per day for women and two for men, each containing around 14 g of ethanol (U.S. Department of Health and Human Services, 2020) [52]. High-risk drinking is typically >7 drinks per week for women and >14 for men. Thus, the threshold used in our study may be considered relatively high compared to other international recommendations. While some studies have highlighted the potential cardioprotective effects of moderate alcohol use, our findings suggest that, in this occupational cohort, alcohol acts predominantly as a risk enhancer—especially in the presence of other unhealthy behaviors. This is consistent with recent meta-analyses questioning the net benefits of alcohol consumption [53,54].

Interestingly, even among individuals with generally healthier profiles—such as those with higher education or adherence to healthy habits—there was a slight but notable increase in atherogenic indices over time. This trend may reflect broader societal changes, such as increased consumption of processed foods, reduced occupational physical activity, and psychosocial stress, despite awareness of cardiovascular health promotion [55].

Our findings are in line with recent results from the Spanish ENRICA study and the French CONSTANCES cohort, both of which highlight strong associations between physical inactivity and dyslipidemia, particularly in working populations [56,57]

The longitudinal data underscore the need for sustained, population-level interventions and suggest that passive health education may be insufficient to counteract structural drivers of risk.

The 2019 guidelines of the European Society of Cardiology (ESC) and the European Atherosclerosis Society (EAS) on dyslipidemia management, along with the 2021 ESC guidelines on cardiovascular disease prevention, emphasize the importance of lipid control and comprehensive cardiovascular risk assessment to reduce atherogenic burden and adverse events [58,59].

A key component is stratifying individuals according to total cardiovascular risk to define appropriate LDL cholesterol (LDL-c) targets. For very high-risk patients (e.g., established ASCVD or diabetes with target organ damage), an LDL-c goal of <1.4 mmol/L (<55 mg/dL) and ≥50% reduction from baseline is recommended. In selected cases with recurrent events, a target of <1.0 mmol/L (<40 mg/dL) may be considered. High-risk individuals should aim for <1.8 mmol/L (<70 mg/dL) and moderate-risk individuals < 2.6 mmol/L (<100 mg/dL).

Beyond LDL-c, the ESC/EAS guidelines highlight the need to address atherogenic dyslipidemia—elevated triglycerides, low HDL-c, and small, dense LDL particles—especially common in individuals with insulin resistance, obesity, or metabolic syndrome. Management includes intensive lifestyle intervention and combination lipid-lowering therapy, such as statins plus ezetimibe or PCSK9 inhibitors.

To support implementation, the SCORE2 and SCORE2-OP tools estimate 10-year cardiovascular event risk by age, sex, and regional mortality data. In 2021, the ESC introduced the SCORE2 and SCORE2-Older Persons (SCORE2-OP) systems to estimate 10-year cardiovascular risk, accounting for age, sex, and region-specific cardiovascular mortality rates. These tools support tailored therapeutic strategies and shared decision-making. In this context, the triglyceride-to-HDL-cholesterol (TG/HDL-C) ratio has gained relevance as a simple and accessible marker of insulin resistance and atherogenic risk. However, its cut-off values differ across populations, highlighting the need for ethnicity- and region-specific thresholds to ensure accurate risk stratification. Applying generalized cut-offs in heterogeneous populations may misrepresent cardiometabolic risk. Therefore, the ESC/EAS guidelines advocate for a personalized, risk-based approach to lipid management, where risk scores and indirect markers like TG/HDL-C can enhance cardiovascular risk assessment and guide more effective preventive strategies.

In light of these findings, the implications for public health and occupational medicine are significant. Interventions aiming to reduce cardiovascular risk should not only target clinical parameters but also address upstream determinants such as social class, educational opportunity, and workplace conditions. Policies that promote physical activity, facilitate access to healthy food, and reduce harmful alcohol consumption—particularly among vulnerable groups—could yield substantial benefits. Moreover, regular monitoring of atherogenic indices in occupational settings could enhance early identification of at-risk individuals and support timely, tailored interventions.

Future research should explore the causal pathways linking social and behavioral factors to atherogenic risk using longitudinal designs and mediation analysis. It would also be valuable to investigate the role of occupational stress, shift work, and environmental exposures—factors not assessed in this study—in modulating lipid profiles. Finally, sex-specific mechanisms warrant further exploration, given the distinct patterns observed in this study and the growing emphasis on personalized medicine.

In conclusion, this study highlights the complex interplay between lifestyle behaviors, sociodemographic factors, and lipid-related cardiovascular risk in a large working population. Our findings emphasize the need for integrated prevention strategies that combine individual-level behavior change with broader structural interventions to address social inequalities and promote cardiovascular health across the lifespan.

## 5. Strengths and Limitations

One of the strengths of this study lies in its large, diverse, and well-characterized population. The workplace-based health surveillance context allowed for uniform data collection and minimized selection bias. Moreover, the inclusion of both cross-sectional and longitudinal analyses adds depth to our understanding of risk evolution and its behavioral and social determinants. The use of multiple atherogenic indices—rather than relying solely on individual lipid parameters—provides a nuanced perspective on cardiovascular risk stratification.

Nevertheless, several limitations must be acknowledged. First, the observational nature of the study precludes causal inference. Although robust associations were identified, it is possible that unmeasured confounding variables—such as genetic predisposition or unreported medication use—may have influenced the results. Second, lifestyle data were self-reported and may be subject to recall or social desirability bias. Third, while the occupational cohort reflects a broad range of socioeconomic backgrounds, the findings may not be fully generalizable to unemployed individuals, retirees, or institutionalized populations.

Data on chronic diseases, pharmacological treatments, and medication adherence were not available, which may have influenced lipid profiles. We acknowledge this as a limitation affecting the interpretation of long-term risk evolution

## 6. Conclusions

This large-scale occupational cohort study provides robust evidence that sociodemographic and lifestyle factors are independently and significantly associated with atherogenic lipid profiles and the presence of atherogenic dyslipidemia. Age, male sex, low educational attainment, and lower social class were consistently linked to higher cardiovascular risk, as were modifiable behaviors such as smoking, physical inactivity, poor adherence to the Mediterranean diet, and alcohol consumption. Importantly, longitudinal data revealed a concerning increase in the prevalence of high-risk atherogenic indices over the past decade, particularly among individuals with unhealthy behaviors and those from socioeconomically disadvantaged backgrounds.

The findings underscore the need for multifaceted public health strategies that integrate behavioral interventions with efforts to reduce social inequalities in cardiovascular risk. In occupational settings, routine monitoring of atherogenic indices could serve as a valuable tool for early risk detection and targeted health promotion. Promoting sustained physical activity, healthy dietary patterns, and reduced alcohol intake should be central components of cardiovascular prevention programs, particularly among working-age populations.

Future research should further elucidate causal pathways and explore the effectiveness of workplace-based interventions in mitigating lipid-related cardiovascular risk. Ultimately, addressing both the social and behavioral determinants of atherogenic dyslipidemia will be essential for reducing the burden of cardiovascular disease and promoting health equity.

## Figures and Tables

**Table 1:** Characteristics of the population.

	Men *n* = 83,282	Women *n* = 56,352	
	Mean (SD)	Mean (SD)	*p*-Value
Age (years)	41.4 (10.7)	40.1 (10.4)	0.089
Height (cm)	173.8 (7.1)	161.2 (6.5)	<0.001
Weight (kg)	83.2 (14.6)	66.3 (13.9)	<0.001
BMI (kg/m^2^)	27.5 (4.5)	25.5 (5.3)	<0.001
Systolic blood pressure (mmHg)	126.2 (15.9)	115.6 (15.7)	<0.001
Diastolic blood pressure (mmHg)	76.6 (10.9)	71.1 (10.7)	<0.001
Total cholesterol (mg/dL)	199.6 (38.6)	194.6 (36.9)	<0.001
HDL cholesterol (mg/dL)	50.0 (7.7)	54.7 (9.2)	<0.001
LDL cholesterol (mg/dL)	122.6 (37.4)	121.5 (37.1)	<0.001
Triglycerides (mg/dL)	133.8 (95.6)	90.8 (49.7)	<0.001
Glucose (mg/dL)	93.0 (25.4)	86.8 (18.1)	<0.001
	**%**	**%**	***p*-Value**
<30 years	15.1	18.0	<0.001
30–39 years	29.6	31.0	
40–49 years	30.2	30.3	
50–59 years	20.9	17.7	
60–69 years	4.2	3.0	
Underweight	0.5	2.7	<0.001
Normalweight	30.3	52.3	
Overweight	43.0	26.9	
Obesity	26.2	18.1	
Social class I	7.5	13.6	<0.001
Social class II	23.8	32.1	
Social class III	68.7	54.1	
Elementary school	66.4	48.1	<0.001
High school	26.9	40.0	
University	6.7	11.9	
Non-smokers	66.8	67.9	<0.001
Smokers	33.2	32.1	
No physical activity	62.4	51.4	<0.001
Yes physical activity	37.6	48.6	
No Mediterranean diet	65.8	52.8	<0.001
Yes Mediterranean diet	34.2	47.2	
No alcohol consumption	67.3	84.4	<0.001
Yes alcohol consumption	32.7	15.6	

**Table 2:** Mean values of different atherogenic scales according sociodemographic variables and healthy habits by gender.

		TC/HDL-c	LDL-c/HDL-c	TG/HDL-c
Men	*n*	Mean (SD)	Mean (SD)	Mean (SD)
<30 years	12,558	3.3 (0.9)	1.9 (0.8)	1.9 (1.4)
30–39 years	24,648	3.9 (1.1)	2.4 (0.9)	2.5 (2.3)
40–49 years	25,178	4.3 (1.2)	2.7 (1.0)	3.1 (2.8)
50–59 years	17,370	4.6 (1.2)	2.9 (1.0)	3.3 (2.2)
60–69 years	3528	4.6 (1.2)	3.0 (1.1)	3.4 (2.8)
Social class I	6234	4.0 (1.1)	2.5 (1.0)	2.6 (2.1)
Social class II	19,856	4.1 (1.1)	2.6 (0.9)	2.8 (2.3)
Social class III	57,192	4.1 (1.3)	2.6 (1.1)	2.9 (2.6)
Elementary school	55,306	4.2 (1.2)	2.7 (1.0)	3.0 (2.7)
High school	22,408	4.1 (1.2)	2.6 (1.0)	2.8 (2.4)
University	5568	4.0 (1.1)	2.5 (1.0)	2.6 (2.1)
Non-smokers	55,618	4.1 (1.1)	2.5 (1.0)	2.7 (2.2)
Smokers	27,664	4.2 (1.4)	2.6 (1.1)	3.1 (3.0)
No physical activity	51,984	4.5 (1.3)	2.8 (1.1)	3.6 (2.9)
Yes physical activity	31,298	3.4 (0.7)	2.1 (0.7)	1.6 (0.6)
No Mediterranean diet	54,792	4.5 (1.3)	2.8 (1.1)	3.5 (2.8)
Yes Mediterranean diet	28,490	3.4 (0.7)	2.1 (0.7)	1.6 (0.7)
No alcohol consumption	56,022	3.9 (1.1)	2.4 (1.0)	2.4 (1.9)
Yes alcohol consumption	27,260	4.5 (1.3)	2.8 (1.1)	3.8 (3.2)
**Women**	***n***	**Mean (SD)**	**Mean (SD)**	**Mean (SD)**
<30 years	10,110	3.1 (0.9)	1.8 (0.8)	1.4 (0.8)
30–39 years	17,460	3.4 (1.0)	2.1 (0.9)	1.6 (0.9)
40–49 years	17,094	3.8 (1.0)	2.5 (0.9)	1.8 (1.2)
50–59 years	9984	4.3 (1.1)	2.9 (1.0)	2.2 (1.4)
60–69 years	1704	4.4 (1.1)	2.9 (1.0)	2.3 (1.2)
Social class I	7632	3.3 (1.0)	2.0 (0.9)	1.5 (1.0)
Social class II	18,112	3.7 (1.0)	2.3 (0.9)	1.7 (1.2)
Social class III	30,608	3.8 (1.1)	2.4 (1.0)	1.8 (1.1)
Elementary school	27,086	3.8 (1.1)	2.4 (1.0)	1.8 (1.2)
High school	22,574	3.6 (1.1)	2.3 (1.0)	1.7 (1.1)
University	6692	3.3 (1.0)	2.0 (0.9)	1.5 (1.1)
Non-smokers	38,252	3.6 (1.1)	2.3 (1.0)	1.7 (1.1)
Smokers	18,100	3.7 (1.1)	2.4 (0.9)	1.8 (1.2)
No physical activity	28,962	4.2 (1.1)	2.7 (1.0)	2.2 (1.4)
Yes physical activity	27,390	3.2 (0.7)	1.9 (0.7)	1.3 (0.4)
No Mediterranean diet	29,764	4.1 (1.1)	2.7 (1.0)	2.1 (1.4)
Yes Mediterranean diet	26,588	3.2 (0.7)	1.9 (0.7)	1.3 (0.5)
No alcohol consumption	47,536	3.6 (1.0)	2.3 (0.9)	1.6 (0.9)
Yes alcohol consumption	8816	4.2 (1.1)	2.7 (1.0)	2.5 (1.8)

**Table 3:** Prevalence of high values of different IR scales according sociodemographic variables and healthy habits by gender.

		TC/HDL-c Moderate–High	LDL-c/HDL-c High	TG/HDL-c High	AD
Men	*n*	%	%	%	%
<30 years	12,558	3.8	3.3	11.2	1.9
30–39 years	24,648	11.2	9.5	22.2	5.2
40–49 years	25,178	22.0	17.8	34.7	11.3
50–59 years	17,370	31.1	25.5	40.4	20.1
60–69 years	3528	31.4	25.7	42.1	27.5
Social class I	6234	18.1	17.4	24.5	8.4
Social class II	19,856	18.7	16.5	29.3	10.0
Social class III	57,192	18.9	15.1	29.4	11.1
Elementary school	55,306	20.0	17.7	30.9	13.0
High school	22,408	19.6	15.5	28.5	9.8
University	5568	17.6	14.7	25.7	8.9
Non-smokers	55,618	17.6	14.4	27.6	8.8
Smokers	27,664	19.8	16.6	31.6	14.3
No physical activity	51,984	28.6	23.3	45.5	15.7
Yes physical activity	31,298	1.3	1.5	1.5	2.1
No Mediterranean diet	54,792	27.1	21.7	42.7	15.0
Yes Mediterranean diet	28,490	2.2	2.4	2.6	2.2
Nn alcohol consumption	56,022	13.6	12.3	20.2	4.3
Yes alcohol consumption	27,260	28.1	20.9	47.1	11.1
**Women**	***n***	**%**	**%**	**%**	**%**
<30 years	10,110	6.5	8.9	4.1	1.6
30–39 years	17,460	12.7	16.4	5.6	3.1
40–49 years	17,094	22.1	26.3	9.1	6.3
50–59 years	9984	38.4	43.4	17.3	16.1
60–69 years	1704	39.8	44.8	21.8	27.3
Social class I	7632	12.3	15.6	10.4	3.0
Social class II	18,112	18.7	26.4	8.3	5.6
Social class III	30,608	22.4	22.6	4.8	8.4
Elementary school	27,086	23.0	27.2	10.6	8.7
High school	22,574	18.4	22.1	8.4	5.6
University	6692	11.9	15.2	4.3	2.8
Non-smokers	38,252	18.8	21.9	8.7	6.2
Smokers	18,100	20.3	24.6	9.5	7.0
No physical activity	28,962	35.3	39.2	17.4	12.2
Yes physical activity	27,390	3.6	7.3	1.1	1.6
No Mediterranean diet	29,764	33.2	37.2	16.5	11.8
Yes Mediterranean diet	26,588	4.9	8.6	0.8	1.1
No alcohol consumption	47,536	17.1	21.5	5.9	2.6
Yes alcohol consumption	8816	34.6	35.8	25.6	14.2

**Table 4:** Multinomial logistic regression.

	TC/HDL-c Moderate–High	LDL-C/HDL-C High	TG/HDL-c High	Atherogenic Dyslipidemia
	OR (95% CI)	OR (95% CI)	OR (95% CI)	OR (95% CI)
Women	1	1	1	1
Men	0.68 (0.66–0.70)	0.43 (0.42–0.44)	3.76 (3.62–3.89)	1.84 (1.70–1.98)
<30 years	1	1	1	1
30–39 years	1.10 (1.07–1.14)	1.19 (1.15–1.23)	1.06 (1.04–1.09)	1.14 (1.10–1.18)
40–49 years	1.50 (1.40–1.60)	1.62 (1.52–1.72)	1.17 (1.09–1.26)	1.57 (1.43–1.71)
50–59 years	2.50 (2.33–2.68)	2.58 (2.40–2.76)	1.51 (1.41–1.70)	2.03 (1.83–2.24)
60–69 years	4.96 (4.55–5.37)	5.01 (4.59–5.43)	2.08 (1.91–2.27)	2.81 (2.45–3.17)
Social class I	1	1	1	1
Social class II	1.09 (1.06–1.10)	1.10 (1.06–1.14)	1.14 (1.09–1.19)	1.09 (1.05–1.14)
Social class III	1.29 (1.24–1.35)	1.20 (1.16–1.25)	1.31 (1.21–1.32)	1.20 (1.15–1.26)
University	1	1	1	1
High school	1.12 (1.09–1.15)	1.11 (1.08–1.14)	1.13 (1.09–1.17)	1.09 (1.06–1.12)
Elementary school	1.23 (1.19–1.27)	1.29 (1.23–1.35)	1.29 (1.23–1.36)	1.15 (1.12–1.18)
Non-smokers	1	1	1	1
Smokers	1.18 (1.14–1.22)	1.15 (1.12–1.18)	1.16 (1.12–1.20)	1.10 (1.08–1.12)
Yes physical activity	1	1	1	1
No physical activity	13.52 (12.43–14.62)	8.08 (7.52–8.64)	36.23 (32.12–40.35)	16.86 (14.80–18.93)
Yes Mediterranean diet	1	1	1	1
No Mediterranean diet	1.26 (1.20–1.32)	1.22 (1.14–1.30)	1.59 (1.45–1.73)	7.46 (6.70–8.22)
No alcohol consumption	1	1	1	1
Yes alcohol consumption	2.33 (2.15–2.51)	2.19 (2.02–2.36)	1.60 (1.54–1.66)	1.79 (1.70–1.88)

**Table 5:** Longitudinal trends in atherogenic risk by sex.

			TC/HDL-c Moderate–High			LDL-c/HDL-c High			TG/HDL-c High			AD	
		PRE	POST		PRE	POST		PRE	POST		PRE	POST	
Men	*n*	%	%	Difference (%)	%	%	Difference (%)	%	%	Difference (%)	%	%	Difference (%)
<30 years	3645	7.9	8.4	6.9	10.3	11.3	9.9	7.1	7.7	8.8	3.1	3.3	7.1
30–39 years	6933	12.8	14.1	10.3	17.9	20.4	13.7	14.2	16.0	12.5	4.9	5.4	9.9
40–49 years	7013	15.9	18.2	14.5	23.8	28.5	19.8	21.0	25.1	19.7	7.9	8.9	12.8
50–59 years	4952	20.3	24.3	19.8	28.9	35.8	23.9	27.8	33.7	21.4	10.3	12.1	17.5
Social class I	1760	9.9	10.6	7.2	13.8	15.6	12.8	10.9	12.3	12.5	3.8	4.1	7.8
Social class II	5368	11.8	13.1	10.9	19.8	23.1	16.8	16.8	19.5	16.1	4.9	5.5	11.9
Social class III	15,415	15.8	18.6	17.8	28.2	33.9	20.1	24.4	29.4	20.3	7.9	9.3	17.2
Elementary school	14,914	15.9	18.8	18.1	27.7	33.3	20.3	24.1	29.1	20.6	8.0	9.4	17.1
High school	6053	12.0	13.4	11.4	20.2	23.8	17.8	17.0	19.7	16.0	5.1	5.7	11.8
University	1576	9.8	10.9	10.9	14.0	15.5	10.9	11.0	12.3	12.0	3.7	4.0	7.7
Non-smokers	15,122	12.8	15.0	17.5	17.5	20.1	15.1	15.9	17.6	10.9	5.9	6.5	10.9
Smokers	7421	16.9	20.3	19.9	22.9	28.0	22.2	22.4	26.3	17.3	10.8	12.8	18.5
Yes physical activity	8535	4.8	5.0	3.8	6.8	7.3	7.1	5.8	6.1	5.8	2.3	2.5	7.1
No physical activity	14,008	19.7	23.4	18.9	29.9	37.0	23.9	23.1	27.8	20.4	15.8	19.6	23.8
Yes Mediterranean diet	7767	6.1	6.4	4.8	7.7	8.3	8.2	6.9	7.4	7.9	3.5	3.8	8.8
No Mediterranean diet	14,776	17.7	20.9	17.8	25.8	31.1	20.5	20.3	24.6	21.1	10.9	13.1	20.1
No alcohol consumption	15,107	10.8	11.8	8.8	10.1	11.2	10.5	14.8	16.8	13.3	5.8	6.5	11.9
Yes alcohol consumption	7436	16.8	19.3	14.9	23.9	29.6	23.9	26.9	33.5	24.5	10.9	13.1	19.8
**Women**	***n***	**%**	**%**	**Difference (%)**	**%**	**%**	**Difference (%)**	**%**	**%**	**Difference (%)**	**%**	**%**	**Difference (%)**
<30 years	2833	7.9	8.6	8.6	9.1	9.9	8.8	6.1	6.6	7.7	2.1	2.2	6.0
30–39 years	4824	11.8	13.3	12.9	10.7	12.0	11.7	8.8	9.7	10.2	3.6	3.9	8.7
40–49 years	4636	13.9	16.5	18.8	14.9	17.3	15.9	12.1	13.8	13.8	5.8	6.4	10.9
50–59 years	2768	19.2	23.4	22.1	19.9	25.0	25.8	19.3	22.9	18.8	7.3	8.4	14.8
Social class I	1973	8.9	9.5	6.8	10.1	11.1	9.9	7.5	8.3	10.1	3.1	3.3	7.3
Social class II	4920	11.8	13.1	10.7	14.8	16.8	13.8	10.8	12.4	14.5	4.8	5.3	10.1
Social class III	8168	17.8	21.2	18.9	19.8	23.3	17.9	15.8	18.8	18.8	6.2	7.1	14.5
Elementary school	7289	17.6	20.8	18.1	19.5	23.0	18.1	15.3	18.1	18.6	6.0	6.9	14.3
High school	6056	12.0	13.4	11.6	15.1	17.2	13.9	11.0	12.6	14.7	4.9	5.4	10.2
University	1716	9.0	9.6	7.1	10.2	11.2	9.5	7.7	8.5	10.2	4.7	5.0	7.4
Non-smokers	10,236	10.8	12.0	11.1	13.5	15.1	12.1	10.8	11.9	10.3	4.0	4.4	8.8
Smokers	4825	14.8	16.9	14.2	16.2	19.4	19.8	14.9	17.3	16.4	5.9	6.7	13.8
Yes physical activity	7317	3.8	3.9	3.4	4.4	4.6	5.5	4.1	4.3	4.9	1.8	1.9	5.5
No physical activity	7744	15.8	17.5	10.9	15.5	18.7	20.5	18.8	22.1	17.8	9.5	11.0	16.2
Yes Mediterranean diet	7029	4.9	5.1	3.9	5.1	5.4	6.4	4.9	5.2	5.8	2.3	2.4	5.9
No Mediterranean diet	8032	14.1	15.5	10.1	14.1	16.7	18.5	16.1	18.6	15.4	9.0	10.3	14.6
No alcohol consumption	12,750	8.9	9.6	7.7	7.7	8.5	9.9	7.9	8.7	10.2	3.7	4.0	8.4
Yes alcohol consumption	2311	13.9	15.6	12.2	16.9	20.3	20.3	16.8	20.2	20.3	7.8	9.1	17.2

TC Total cholesterol, HDL-c High-density lipoprotein cholesterol. LDL-c. Low-density lipoprotein cholesterol. TG Triglycerides. AD Atherogenic dyslipidemia. PRE = year 2009, POST = year 2019. The formula for calculating the difference is [(POST − PRE)/PRE] as a percentage.

## Data Availability

The dataset generated and analyzed during the current study is securely stored in a protected database hosted by ADEMA-Escuela Universitaria, adhering to all applicable data security protocols. Oversight of data protection is managed by the institution’s designated Data Protection Officer, Dr. Ángel Arturo López González.
